# Lingmao Formula Combined with Entecavir for HBeAg-Positive Chronic Hepatitis B Patients with Mildly Elevated Alanine Aminotransferase: A Multicenter, Randomized, Double-Blind, Placebo-Controlled Trial

**DOI:** 10.1155/2013/620230

**Published:** 2013-08-24

**Authors:** Xiao-Jun Zhu, Xue-Hua Sun, Zheng-Hua Zhou, Shun-Qing Liu, Hua Lv, Man Li, Lu Li, Yue-Qiu Gao

**Affiliations:** ^1^Department of Hepatopathy, Shuguang Hospital, Shanghai University of Traditional Chinese Medicine, 528 Zhangheng Road, Shanghai 201203, China; ^2^Shanghai Traditional Chinese Medicine Clinical Center of Hepatopathy, 528 Zhangheng Road, Shanghai 201203, China; ^3^Center for Clinical Effect Evaluation, Shuguang Hospital, Shanghai University of Traditional Chinese Medicine, 528 Zhangheng Road, Shanghai 201203, China; ^4^Shanghai Key Laboratory of Traditional Chinese Medicine, 528 Zhangheng Road, Shanghai 201203, China

## Abstract

*Objective*. To determine the efficacy and safety of Lingmao Formula combined with entecavir for HBeAg-positive chronic hepatitis B patients with mildly elevated alanine aminotransferase (ALT). *Methods*. 301 patients were randomly assigned to receive Lingmao Formula combined with entecavir (treatment group) or placebo combined with entecavir (control group) for 52 weeks. The outcomes of interest included the reduction of serum HBV DNA level, HBeAg loss, HBeAg seroconversion, ALT normalization, and histological improvement. *Results*. The mean decrease of serum HBV DNA level from baseline and the percentage of patients who had reduction in serum HBV DNA level ≥2 lg copies/mL in treatment group were significantly greater than that in control group (5.5 versus 5.4 lg copies/mL, *P* = 0.010; 98.5% versus 92.6%, *P* = 0.019). The percentage of HBeAg loss in treatment group was 22.8%, which was much higher than a percentage of 12.6% in control group (*P* = 0.038). There was no significant difference between the two groups in histological improvement. Safety was similar in the two groups. *Conclusions*. The combination of Lingmao Formula with entecavir could result in significant decrease of serum HBV DNA and increase of HBeAg loss for HBeAg-positive chronic hepatitis B patients with mildly elevated ALT without any serious adverse events. Clinical trial registration number is ChiCTR-TRC-09000594.

## 1. Introduction

Hepatitis B virus (HBV) is the most common pathogen of chronic liver diseases. According to the data released by World Health Organization (WHO), two billion people are estimated to have been infected with HBV worldwide and 350 million are chronic HBV carriers [1]. HBV-related liver diseases, including hepatitis, cirrhosis, and hepatocellular carcinoma, are responsible for more than one million deaths annually [2]. Recently, an nationwide survey conducted in China reported a HBV infection rate of 7.18% in Chinese population [3, 4]. Accordingly, about 93 million HBV carriers and 20 million patients with chronic hepatitis B (CHB) are estimated in China [5]. Even worse, more than 300,000 Chinese patients died from the HBV-related diseases every year [6]. 

Current guidelines for the management of CHB recommend that the antiviral treatment should be initiated for hepatitis B e antigen (HBeAg) positive patients if the HBV DNA levels are ≥1 × 10^5^ copies/mL and the ALT levels are at least 2-folds than the upper limit of normal (ULN) [7–10]. HBV-infected patients with mildly elevated serum ALT levels (1-2 × ULN) usually show significant necroinflammation and fibrosis when liver biopsy performed [11]. These patients are at similar risk to develop severe complications as compared with those having higher serum ALT levels [12]. EASL (European Association for the Study of the Liver) Clinical Practice Guidelines suggest that patients with mildly elevated serum ALT levels should also be treated when the HBV DNA levels are higher than 1 × 10^5^ copies/mL and liver biopsy disclosed moderate or severe active necroinflammation and/or fibrosis using a standardized scoring system [10]. However, a few studies have demonstrated that HBeAg-positive CHB patients with mildly elevated ALT respond poorly to the treatment with lamivudine, entecavir, or interferon [13–15]. Therefore, it is urgent to develop novel agents or therapy strategies for this specific patient population.

In China, several traditional Chinese herbs have been used to treat CHB for hundreds of decades. Recently, contemporary clinical researches of traditional Chinese medicines for CHB were reviewed. The results showed that some traditional Chinese herbs may enhance the antiviral activities of interferon and lamivudine and improve the liver function of CHB patients [16]. The therapeutic effects of a few of Chinese herbs for CHB patients, such as *astragalus*, *polygonum cuspidatum*, and *phyllanthus urinaria*, have been identified through inhibiting HBV replication [17–19]. The Lingmao Formula, an empirical formula widely prescribed in traditional Chinese medicine for CHB patients, has showed promising inhibition activity of HBsAg and HBeAg secretions *in vitro* in our previous study [20]. However, no additional data regarding the efficacy and safety of the Lingmao Formula is available until now. To determine the therapeutic role of the Lingmao Formula for CHB patients, especially for the CHB patients with mildly evaluated serum ALT levels, a multicenter, randomized, double-blind, and placebo-controlled clinical trial was carried out in the present study. Entecavir was used as a positive control agent and the Lingmao Formula was administrated in combination with entecavir for 52 weeks. The efficacy and safety of the Lingmao Formula combined with entecavir were thoroughly evaluated. 

## 2. Materials and Methods

### 2.1. Study Design

This study was a multicenter, randomized, double-blind, and placebo-controlled clinical trial to evaluate the efficacy and safety of the Lingmao Formula combined with entecavir for HBeAg-positive chronic hepatitis B patients with mildly elevated alanine aminotransferase (ALT). All the patients were treated with entecavir 0.5 mg daily and were randomly assigned to receive the Lingmao Formula or placebo (4.5 g twice daily, oral administration) for 52 weeks, respectively, in treatment and control groups. The Lingmao Formula was composed of Yinyanghuo (*Epimedium brevicornum* Maxim. leaf), Huangqi (*Astragalus membranaceus* (Fisch.) Bge. Var. mongholicus (Bge.) Hsiao. root), Huhuanglian (*Picrorhiza scrophulariiflora* Pennell root), Maozhaocao (*Ranunculus ternatus* Thunb root), Nvzhenzi (*Ligustrum lucidum* Ait. ripe fruit), and Qingpi (*Citrus reticulate* Blanco immature fruit peel). The concentrated granules of the six herbs provided by Jiang Yin Tianjiang Pharmaceutical Co. Ltd. were used in this study. The concentrated granules of the six herbs were packaged individually according to the prescription of the Lingmao Formula, and they were mixed and dissolved by warm water when taken orally. Weights of the six herb concentrated granules in the Lingmao Formula (4.5 g) are Yinyanghuo 0.375 g (lot number: 0903067, amount to original herb of 7.5 g), Huangqi 1.125 g (lot number: 0812028, amount to original herb of 7.5 g), Huhuanglian 0.75 g (lot number: 0904046, amount to original herb of 4.5 g), Maozhaocao 0.75 g (lot number: 0802045, amount to original herb of 7.5 g), Nvzhenzi 0.75 g (lot number: 0812091, amount to original herb of 7.5 g), and Qingpi 0.75 g (lot number: 0811006, amount to original herb of 4.5 g). 

There is a corresponding placebo also in concentrated granule form for each herb in the Lingmao Formula. The placebos were provided by the same company: placebo of Yinyanghuo (lot number: 0903334), placebo of Huangqi (lot number: 0903332), placebo of Huhuanglian (lot number: 0903336), placebo of Maozhaocao (lot number: 0903335) placebo of Nvzhenzi (lot number: 0903333), and placebo of Qingpi (lot number: 0903337). They were composed of food coloring (tartrazine, sunset yellow, and caramel color), bittering agent (sucrose octaacetate), and pharmaceutical excipient (Lactose). Food coloring, bittering agent, and pharmaceutical excipient were conducted in accordance with the Chinese regulation on the management of pharmaceutical excipients and Chinese hygienic standards for uses of food additives. The placebos shared the same package and label as the herb granules. The appearance, taste and solubitity of placebos were similar to the herb granules. They were also taken the same way as the Lingmao Formula. This study was conducted in accordance with the ethics principles of the Declaration of Helsinki and regulation of clinical trials [21]. It was also approved by the Ethical Committee of Shuguang Hospital, Shanghai University of Traditional Chinese Medicine. Written informed consent was obtained from all enrolled patients.

### 2.2. Sample Size Calculation and Randomization

For HBeAg-positive patients with mildly elevated ALT, our previous pilot study showed that HBV DNA was undetectable by PCR assay (HBV DNA < 1000 copies/mL) in 47% of patients when treated by entecavir alone and in 65% of patients treated by both the Lingmao Formula and entecavir. If *α* = 0.05, *β* = 0.20, and a drop-out rate of 20% was assumed, 300 subjects would be required for enrollment into the study. By using a block randomization method, the enrolled patients were assigned into two groups with four patients in one block with a proportion of 1 : 1. The randomization was performed by SAS version 9.0 (SAS Institute, Cary, NC, USA).

### 2.3. Study Population

The inclusion criteria included aged from 18 to 65; hepatitis B surface antigen (HBsAg) was detectable for at least 24 weeks; HBeAg-positive; compensated liver function; mildly elevated serum ALT levels (1-2 × ULN) at least 4 weeks before screening; HBV DNA level was ≥1 × 10^5^ copies/mL by PCR assay at least 4 weeks before screening; untreated with any nucleoside analogues, pegylated interferon *α*, or interferon *α* at least 12 months before screening; and liver-biopsy specimens with a Scheuer necroinflammatory grade of 2 or greater within 12 weeks before randomization. The exclusion criteria included being coinfected with HAV, HCV, HDV, HEV, and HIV; hepatocellular carcinoma; other forms of liver disease; being other severe primary disease or mental disorder disease; treated to decrease the ALT level within three months; and pregnant and lactating women. The rejection and withdrawal criteria were misincluded or misdiagnosed; without any test record; did not finish the trial according to the program; abnormal serum ALT not due to CHB. 

### 2.4. Efficacy Endpoints

The primary endpoints were listed as follows (1) Virological response: HBV DNA was quantified by real-time PCR assay and the lowest limit of quantification was set at 1 × 10^3^ copies/mL. The extent of HBV DNA change and the rate of decrease in serum HBV DNA of >2 lg copies/mLwere computed. (2) Serological response: the loss rates of HBeAg and HBsAg or the seroconversion rates of HBeAg and HBsAg were, respectively, calculated. (3) Biochemical response: the rate of ALT normalization between the two groups was analyzed. (4) Histological response: positive response was defined as that necroinflammatory was improved by at least one grade in the Scheuer necroinflammatory grade with no worsening in the fibrosis stage, or fibrosis was improved by at least one grade in the Scheuer fibrosis stage with no worsening in the necroinflammatory grade. 

### 2.5. Resistance and Safety Measurements

A resistance analysis was undertaken through identifying the HBV polymerase substitutions which were associated with reduced susceptibility to entecavir. Serum samples from patients at baseline, patients with viral breakthrough, and patients having more than 1 × 10^3^ copies/mL of HBV DNA at week 52 were obtained and analyzed. HBV DNA was quantified by using real-time PCR method and the genotype of HBV DNA polymerase was sequenced. 

Patients were advised to report any change in health. All adverse events were documented by the physicians. Electrocardiogram, complete blood count, urine analysis, serum creatinine, and urea nitrogen were also performed before enrollment and at week 52. The primary safety endpoint was the proportion of patients who discontinued the study medication because of clinical or laboratory-determined adverse events. Other safety evaluations included analyses of adverse events, serious events, and death.

### 2.6. Statistical Analysis

Statistical analyses were performed using SPSS statistics software (version 17.0, IBM Inc., NY, USA). Continuous variables were described by means ± standard deviation (SD). Comparison of continuous variables was analyzed by *t*-test. Categorical variables were described by rate and percentage. Comparison of categorical variables was conducted by Pearson's chi-square test or Fisher's exact test. All *P* values were two-sided with significance level at 0.05. 

## 3. Results

### 3.1. Study Population

**Figure 1:**
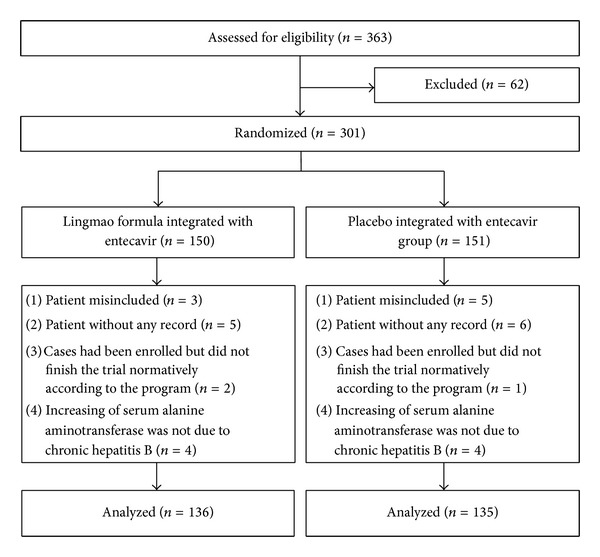
Flow diagram of the randomized clinical trial.

From December 2008 to March 2011, a total of 363 patients were recruited from 12 Chinese hospitals. After 62 patients were excluded, 301 eligible patients were randomly assigned into treatment group or control group in a double-blinded manner. One hundred and fifty patients were treated with the Lingmao Formula and entecavir, and the other 151 patients were treated with placebo and entecavir. In a double-blinded manner, 271 patients were received (136 in the treatment group and 135 in the control group). Among them, 41 patients in the treatment group and 39 patients in the control group had adequate liver biopsy specimens for histopathological evaluation at both baseline and trial end. The baseline characteristics of these patients in the two groups are shown in Table 1 and no difference were found between the two groups. Flow diagram of the trial is show in Figure 1.

### 3.2. Virologic Response

As shown in Table 2, the mean decrease of serum HBV DNA level from baseline in treatment group was significantly greater than that in control group (5.5 versus 5.4 lg copies/mL, *P* = 0.010) at week 52. Also, the percentage of reduction in serum HBV DNA level ≥ 2 lg copies/mL in treatment group was significantly higher than that in control group (98.5% versus 92.6%, *P* = 0.019). HBV DNA was undetectable by PCR assay in 68.4% of patients in the treatment group at week 52 and no difference was found when compared to that in the control group (67.4%, *P* = 0.897).

### 3.3. Serologic Response

As listed in Table 2, the rate of HBeAg loss in treatment group was 22.8%, which was much higher than the rate of 12.6% in control group (*P* = 0.038). At week 52, both undetectable HBV DNA and HBeAg loss were identified in 29 patients of the treatment group (21.3%) and 12 of the control group (8.9%, *P* = 0.006). However, the rates of HBeAg seroconversion between the two groups are not significantly different (14.0% versus 11.1%, *P* = 0.583). At week 52, both undetectable HBV DNA and HBeAg seroconversion were identified in 19 patients of the treatment group (14.0%) and 11 of the control group (8.1%, *P* = 0.175). HBsAg loss only occurred in two patients in treatment group (1.5%) and only one in control group (0.7%).

### 3.4. Biochemical Response

The percentage of ALT normalization in the treatment group was slightly lower than that in the control group and no significant difference was found between them (77.9% versus 78.5%,* P *= 1.000; Table 2). 

### 3.5. Histological Response

The histopathological evaluation showed that 53.7% of patients in treatment group and 33.3% of patients in control group achieved an improved necroinflammatory (*P* = 0.076; Table 2). Similarly, 41.5% of patients in the treatment group and 35.9% of patients in the control group underwent an improved fibrosis (*P* = 0.652, Table 2). 

### 3.6. Resistance

No patient in the study had virologic rebound (an increase of HBV DNA levels from nadir of ≥1 lg copies/mL by PCR assay) during the trial. Genotypic resistance occurred in only one patient in control group at week 52, who had mutations in the L180 M, M250V, and M204I. Elevation in alanine aminotransferase was not observed in this patient. 

### 3.7. Safety and Adverse Events

As shown in Table 3, the frequency of adverse events during the whole trial was similar between the two groups. The most frequent adverse events were upper abdominal pain, diarrhea, fatigue, nausea, dyspepsia, dizziness, itching, headache, and cough, and most of them were of mild grade. There was no serious adverse event in the two groups. In treatment group, one patient discontinued because of an increase in ALT and the other patient discontinued because of mild diarrhea. In control group, one patient also discontinued due to an increase in ALT. None of patients in the two groups underwent hepatic decompensation. Additionally, there were no significant changes in electrocardiogram, complete blood count, urine analysis, serum creatinine, and urea nitrogen prior and after drug administration.

## 4. Discussion

Current management guidelines for CHB patients with high viral load emphasize that antiviral treatment should be initiated once serum ALT exceeds 2 × ULN. But for patients with a mildly elevated ALT, antiviral treatment is recommended to be initiated only in patients with biopsy-confirmed moderate/severe inflammation or significant fibrosis [7–10]. Previous studies have demonstrated that significant pathological changes also occur though ALT is less than 2 × ULN [11, 22, 23]. Our study showed that 83.3% of the Chinese HBeAg-positive CHB patients with mildly elevated ALT had moderate or severe active necroinflammation [24]. There is an emerging opinion that the original definition of normal or healthy ALT is too high [25, 26]. It is possible that stringent adherence to the serum ALT threshold of 2 × ULN could exclude a proportion of patients who might benefit from the antiviral treatment. Therefore, ALT is not reliable and biopsy is necessary for the patients with serum ALT levels between 1 and 2 × ULN.

In our study, the Lingmao Formula combined with entecavir shows significant superiority to placebo combined with entecavir in terms of the rate of decrease in serum HBV DNA of >2 lg copies/mL and the mean reduction of HBV DNA. Similarly, the combination administration was superior to entecavir alone in HBeAg loss. The rate of HBeAg loss increased by 10.2% in treatment group compared to control group. Taken together, the Lingmao Formula enhances the antiviral activity of entecavir. 

Arresting or reversing liver damage is a principal goal in the management of CHB. Histopathological evaluation of the liver tissue remains the definitive method of assessing the disease progression or the drug response for CHB patients. In this study, part of the enrolled patients underwent histopathological evaluation concerning their live damage prior and after trial. The results showed that the Lingmao Formula integrated with entecavir could improve the necroinflammatory and fibrosis for CHB patients though no significant difference was detected between the two groups. Particularly, the improvement of necroinflammatory in the treatment group was higher than that in the placebo integrated with entecavir group by nearly 20%. More sample size of biopsy specimens in further studies is warranted to clarify the superiority of the Lingmao Formula combined with entecavir for CHB patients. 

For HBeAg-positive patients with mildly elevated ALT, entecavir shows lower response in histological improvement, loss of serum HBV DNA, and HBeAg seroconversion than for those with ALT greater than 2 × ULN [14]. When HBeAg-positive patients with mildly elevated ALT receive antiviral therapy, they usually need longer treatment period than those with ALT levels greater than 2 × ULN. The longer treatment period means the increase of the care cost. The economic burden of CHB on national healthcare systems is even more pronounced in China. Li and colleagues reported that CHB accounted for about 4% of the national healthcare expenditure in Hong Kong [27]. The endpoints of antiviral treatment for HBeAg-positive patients are disappearance of serum HBV DNA and either HBeAg loss or HBeAg seroconversion. The undetectable HBV DNA and HBeAg loss increased by 12.4% in treatment group compared to control group. Obviously, the integrative treatment might bring a more optimal cost-effectiveness ratio than entecavir alone for CHB patients. 

 Longer treatment period also means the increased incidence of drug resistance. Preliminary data suggest that the rate of entecavir resistance remained at 1.2% in nucleoside-naive patients after up to 5 years of treatment [28]. In the present study, one patient was found to represent resistant genotype in entecavir group. No viral resistance was observed in the Lingmao Formula with entecavir group at week 52. The potential for reducing the rate of drug resistance in the Lingmao Formula with entecavir group should be monitored for longer time. 

When the safety is concerned, the Lingmao Formula integrated with entecavir showed similar tolerability profile with placebo combined with entecavir. A 52-week continued treatment of the Lingmao Formula integrated with entecavir is well tolerated for the HBeAg-positive patients with mildly elevated ALT.

## 5. Conclusions

This study showed that the Lingmao Formula combined with entecavir had a significant antiviral priority to entecavir alone for HBeAg-positive CHB patients with mildly elevated ALT. Together with its safety profile, our data suggests that the Lingmao Formula integrated with entecavir could be considered as an alternative therapy for CHB patients.

## Figures and Tables

**Table 1:** Demographic and baseline characteristics of the patients.

Characteristics	Treatment group (*n* = 136)	Control group (*n* = 135)	*P* value
Age (years)^†^	33.5 ± 10.1	32.8 ± 9.1	0.346
Male	104 (76.5)	104 (77.0)	1.000
Height (cm)^†^	169.1 ± 6.6	168.9 ± 6.1	0.516
Weight (kg)^†^	65.0 ± 10.0	65.5 ± 10.7	0.824
Body mass index (kg/m^2^)^†^	22.7 ± 2.8	22.9 ± 3.0	0.775
Course of illness (years)^†^	7.7 ± 6.1	6.9 ± 5.8	0.450
HBV DNA (lgcopies/mL)^†^	7.2 ± 1.2	7.3 ± 1.4	0.456
Grade of necroinflammatory*			0.600
G2	28 (68.3)	29 (74.4)	
G3	10 (24.4)	9 (23.1)	
G4	3 (7.3)	1 (2.6)	
Stage of fibrosis*			0.621
S0	0 (0.0)	1 (2.6)	
S1	12 (29.3)	7 (17.9)	
S2	17 (41.5)	19 (48.7)	
S3	9 (22.0)	10 (25.6)	
S4	3 (7.3)	2 (5.1)	
Prior resistance by genotypic analysis*	0 (0)	0 (0)	1.000

*A total of 80 patients had adequate baseline and liver-biopsy specimens at week 52 (41 patients in treatment group and 39 in control group). Values in parentheses are percentages unless indicated otherwise; ^†^value was presented as mean ± standard deviation.

**Table 2:** Histological, virologic, serologic, and biochemical response at week 52.

End points	Treatment group (*n* = 136)	Control group (*n* = 135)	*P* value
Necroinflammatory improvement*	22 (53.7)	13 (33.3)	0.076
Fibrosis improvement*	17 (41.5)	14 (35.9)	0.652
HBV DNA <1000 copies/mL by PCR assay	93 (68.4)	91 (67.4)	0.897
≥2 lg copies/mL reduction in HBV DNA compared to baseline	134 (98.5)	125 (92.6)	0.019
Mean change in HBV DNA from baseline by PCR assay (lg copies/mL)^†^	5.5 ± 1.6	5.4 ± 1.9	0.010
HBeAg loss	31 (22.8)	17 (12.6)	0.038
Undetectable HBV DNA and HBeAg loss	29 (21.3)	12 (8.9)	0.006
HBeAg seroconversion	19 (14.0)	15 (11.1)	0.583
Undetectable HBV DNA and HBeAg seroconversion	19 (14.0)	11 (8.1)	0.175
HBsAg loss	2 (1.5)	1 (0.7)	1.000
ALT normalization	106 (77.9)	106 (78.5)	1.000

*A total of 80 patients had adequate baseline and liver-biopsy specimens at week 52 (41 patients in treatment group and 39 in control group). Values in parentheses are percentages unless indicated otherwise; ^†^value was presented as mean ± standard deviation.

**Table 3:** Accumulative adverse events during the 52 weeks of treatment.

Adverse event	Treatment group (*n* = 136)	Control group (*n* = 135)	*P* value
Fatigue	28 (20.6)	25 (18.5)	0.760
Upper abdominal pain	21 (15.4)	19 (14.1)	0.864
Diarrhea	24 (17.6)	22 (16.3)	0.872
Nausea	21 (15.4)	22 (16.3)	0.869
Dyspepsia	33 (24.3)	35 (25.9)	0.781
Dizziness	12 (8.8)	15 (11.1)	0.550
Itching	24 (17.6)	29 (21.5)	0.447
Headache	20 (14.7)	16 (11.9)	0.592
Cough	8 (5.9)	9 (6.7)	0.808
Serious adverse event	0 (0)	0 (0)	
Discontinuation due to adverse event	2 (1.4)	1 (0.7)	1.000
Death	0 (0)	0 (0)	

Values in parentheses are percentages unless indicated otherwise.
